# The Association between Age, Comorbidities and Use of Radiotherapy in Women with Breast Cancer: Implications for Survival

**DOI:** 10.3390/medicines5030062

**Published:** 2018-06-25

**Authors:** Jimmy T. Efird, Sharyn Hunter, Sally Chan, Sarah Jeong, Susan L. Thomas, Charulata Jindal, Tithi Biswas

**Affiliations:** 1 Centre for Clinical Epidemiology and Biostatistics (CCEB), School of Medicine and Public Health, The University of Newcastle (UoN), Newcastle 2308, Australia; charu.jindal@uon.edu.au; 2 Priority Research Centre for Generational Health and Ageing (PRCGHA), School of Medicine and Public Health, the University of Newcastle (UoN), Newcastle 2308, Australia; 3 School of Nursing and Midwifery, the University of Newcastle (UoN), Callaghan 2308, Australia; sharyn.hunter@newcastle.edu.au (S.H.); sally.chan@newcastle.edu.au (S.C.); sarah.jeong@newcastle.edu.au (S.J.); susan.thomas@newcastle.edu.au (S.L.T.); 4 Department of Radiation Oncology, University Hospitals, Case Western Reserve University, Cleveland, OH 44106, USA; tithi.biswas@uhhospitals.org

**Keywords:** breast cancer, comorbidities, older women, radiotherapy, survival

## Abstract

**Background:** Radiotherapy (RT) plays an important role in the management and survival of patients with breast cancer. The aim of this study was to examine the association between age, comorbidities and use of RT in this population. **Methods:** Patients diagnosed with breast cancer from 2004–2013 were identified from the American College of Surgeons National Cancer Database (NCDB). Follow-up time was measured from the date of diagnosis (baseline) to the date of death or censoring. Adjusted hazard ratios (aHR) and 95% confidence intervals (95% CI) were used as the measure of association. **Results:** Independently of comorbidities and other important outcome-related factors, patients >65 years of age who received RT survived significantly longer than those who did not receive RT (aHR = 0.53, 95% CI = 0.52–0.54). However, as women aged, those with comorbidities were less likely to receive RT (adjusted *p*-trend by age < 0.0001). **Conclusions:** The development of decision-making tools to assist clinicians, and older women with breast cancer and comorbidities, are needed to facilitate personalized treatment plans regarding RT. This is particularly relevant as the population ages and the number of women with breast cancer is expected to increase in the near future.

## 1. Introduction

Breast cancer is a disease of aging, predominately affecting women >65 years [1,2]. Over the next generation, the incidence of breast cancer in older women is expected to rise, influencing health service planning and delivery [3]. While there has been progress in the early detection and effective treatment of breast cancer, these gains are less evident in older women [1,4]. One out of two deaths from breast cancer occurs among women in this age group [3].

Radiotherapy (RT) is an important treatment modality for patients with breast cancer [5,6,7,8]. However, misperceptions exist about its appropriateness and efficacy in older women [1,2,3,9,10,11,12]. These include slower disease progression, less aggressive tumors and an assumption that mortality will be attributed to age-related comorbidities [3]. This paper explores the hypothesis that women >65 years of age with comorbidities are less likely to receive RT than women in younger age groups. 

## 2. Materials and Methods

### 2.1. Data Source

Over 1500 Commission on Cancer (CoC) accredited programs in the United States provide information on incident cancer cases to the National Cancer Database (NCDB) [13]. This database, which is maintained by the American College of Surgeons, is the largest cancer registry in the world and contains nearly 10 million cases. Participant hospitals must fulfil 35 benchmark criteria, applicable to the delivery of cancer care, to be accredited by the CoC. Every three years, hospitals are re-evaluated for adherence to these standards. Records in NCDB are de-identified. This study was considered exempt by the institutional review board (IRB) at the recipient NCDB member facility (Code of Federal Regulations 45 part 46.101(b)). 

### 2.2. Eligibility

Patients who underwent surgical resection for primary, invasive breast cancer from 2004–2013 were included in the study. All tumors were pathologically confirmed. They were excluded if their tumors were in situ or advanced clinical stage IV. Patients receiving RT also were excluded if their dose was not within the range of 4000–6000 centigray (cGy) or if the primary target was outside the breast, chest wall or lymph nodes. Sarcoma, lymphoma, and leukaemia histologies were not considered in the current analysis. 

### 2.3. Definitions

Clinical and pathological stage were coded and assessed by each CoC facility using the American Joint Committee on Cancer (AJCC) TNM (Tumor, Nodes and Metastasis) system [14]. The majority of patients were staged according to the 6th and 7th editions of AJCC. Data were not converted from the lower TNM editions. Instead, a sensitivity analysis was performed by stratifying data by year of diagnosis, with the cut-off value based on the year when the 7^th^ edition of AJCC was introduced (i.e., 2010). Payor type consisted of mutually exclusive categories and did not allow for multiple entries for an individual patient (e.g., Medicare with supplemental insurance). Racial identity was self-reported. Age groups were categorized as <45, 45–65 and >65 years.

Breast cancers were subtyped into four groups on the basis of hormone receptor (HR) and epidermal growth factor receptor (HER_2_) status. Comorbidities were categorized using the Charlson (Deyo) Comorbidity Index (CCI). In this index, comorbid conditions are mapped using up to 10 International Classification of Diseases, 9th Revision, Clinical Modification (ICD-9-CM) secondary diagnosis codes and assigned a weighted score between 0 and 25. Patients with no comorbidities were assigned a score of 0. In NCDB, the highest score of 2 is a truncated value corresponding to the presence of multiple comorbidities [13,15]. The surgical procedure of the primary site was dichotomized based on NCDB surgical codes as breast conserving surgery (BCS)/partial mastectomy (20–24) and mastectomy (30, 40–46, 50–56, 60–67, 70–72, 80). 

### 2.4. Statistical Analysis

Categorical variables were denoted as frequencies and percentages, while continuous variables were reported as medians and interquartile ranges (IQR). Statistical significance for categorical variables was tested using the chi-square (χ^2^) procedure and the Kruskal–Wallis H test for continuous variables. Trends across categories were assessed using a Cochran-Armitage trend test with *p* values computed using a likelihood ratio procedure [16]. Follow-up time was measured from the date of diagnosis (baseline) to the date of death or censoring. Adjusted odds ratios (aOR) and hazard ratios (aHR) were estimated using logistic and Cox regression models, respectively. Survival probabilities at 2, 5, 8, and 10 years were computed using the product-limit method. The parallel-hazards assumption was not violated in our models. 

Unless indicated otherwise, the reference group for binary variables was the complement of the indicated category. Other variables were categorized according to NCDB definitions. A multistage-iterative expectation-maximization (EM) algorithm was used to account for missing values [17]. Statistical significance was defined as *p* ≤ 0.05. SAS Version 9.4 (SAS Institute, Cary, NC, USA) was used to analyse the data. 

## 3. Results

The median age of women at diagnosis was 59 years (*n* = 980,381; IQR = 20) (Table 1). Over half of patients lived more than 9 miles from their treatment facility, which in most cases was a comprehensive community cancer centre (49%). The majority of women were white (84%), had private health insurance (56%), and presented with clinical stage I-II disease (69%). Among women >65 years of age, 75% had well or moderately differentiated tumors and only 16% had lymph node invasion. Most tumors in this age group were ≤2 cm (67%). Approximately 17% of older women had the more aggressive triple negative breast cancer (HR^−^/HER_2_^−^). 

The majority of patients received RT (61%), with a median dose of 5000 cGy (IQR = 440 cGy) (Table 2). Among those receiving radiation, treatment was administered prior to surgery in ~1.8% of cases. Women >65 years, independent of biologic subtype, were less likely to receive RT (53%) and to have their lymph nodes treated (17%). Compared with younger women ≤65, they were more likely to receive lower doses of radiation, with a greater percentage falling within the 4000–4500 cGy range. This older age group also was less likely to receive neoadjuvant chemotherapy (NACT). When they did receive NACT, their pathologic complete response (pCR) rate was the lowest of all age groups (11%). Fewer women >65 years received mastectomy (36%) and chemotherapy (24%). 

As women aged, those with more comorbidities were less likely to receive RT (Table 3). For example, the aOR associated with not receiving RT (corresponding to CCI level II vs level 0), increased across age groups (Age <45 years: aOR = 1.1, 95% CI = 0.91–1.3; Age 45–65 years: aOR = 1.3, 95% CI = 1.26–1.41; Age >65 years: aOR = 1.6, 95% CI = 1.5–1.7; adjusted *p*-trend by age <0.0001). Women >65 years with CCI = 2 were 2.2-fold (95% CI = 2.1–2.3) less likely to receive RT than those who were <45 with CCI = 0.

In addition to aOR differences, a linear trend with respect to CCI was observed within the age categories of 45–65 and >65 years (adjusted *p* < 0.0001); as hypothesized, this trend was less evident among women aged <45 years (adjusted *p* = 0.020). A noticeably higher, 1.9-fold aOR (CCI level II vs. level 0; 95% CI = 1.5–1.9) was observed among women aged >65 with HR^−^/HER_2_^+^ tumors compared with other hormone receptor categories. In contrast, women in the same age group with tumor type HR^−^/HER_2_^−^ had a 1.3-fold aOR (95% CI = 1.2–1.5). Even when women aged >65 years with comorbidities (CCI, I and II combined) did receive RT, they were more likely to receive lower doses in the 4000–4500 cGy range than younger women with comorbidities (>65 years, 28%; 45–65 years, 22%; <45 years, 17%; adjusted *p*-trend < 0.0001). Among patients >65 years, tumors identified as originating in the left breast were similarly distributed between those who received (51%) and did not receive RT (51%). 

Adjusted survival probabilities (2, 5, 8, and 10 years) for older women who received radiation, were consistently greater than those not receiving RT (aHR = 0.53, 95% CI = 0.52–0.54) (Table 4). For example, the probability of surviving 5 years was 91% among women who received radiation, compared with 83% for those who did not receive radiation. Independent of comorbidities and other important outcome-related factors, RT was associated with improved overall survival (OS) for all biologic subtypes. 

## 4. Discussion

Comorbidities are common among older women with breast cancer, although the two do not always coexist [11,18]. A common belief or misperception among health care providers is that breast cancer progresses more slowly in older women and the patient is more likely to die from comorbidities [3,19]. Life expectancy may be underestimated even though many older women with comorbidities are living longer due to better management of their conditions [19,20]. While age and comorbidities should be considered, they should never alone be a barrier to standard treatment. This includes the benefits of RT for older women [19,20,21,22,23].

In this study, as women aged, those with more comorbidities were less likely to receive RT. Furthermore, overall survival was consistently greater for older women who received RT.

### 4.1. RT Survival and Local Control

Similar to our findings, several studies have reported that older women are less likely to receive RT than younger women and when RT was administered to this group they generally had better local and regional control and improved survival [24,25,26,27,28,29]. An analysis of 4836 women (50–89 years) reported that as women aged, RT was more likely to be omitted (26% for women aged ≥75 years compared with 7% of the time for women aged 50–64 years). RT omission was associated with significantly reduced local control, breast cancer specific survival (BCSS) and OS. Women aged ≥75 had lower 5-year OS and BCSS when RT was omitted [28]. 

Another study of 44,731 patients with triple negative breast cancer (TNBC) (aged 19–90 years) found that RT was associated with improved OS for women of all ages. Approximately 40% of patients aged 76–80 years, who might have benefitted from RT, did not receive it (although the median expected survival was greater than 10 years for this age group) [24]. Our findings are again confirmed by a large study of 27,399 women with early stage breast cancer, many who had one or more comorbidities, reported increasing survival with age among those receiving RT [27]. However, RT was not associated with improved BCSS and OS in a longitudinal study of 636 women aged ≥70 who received BCS and Tamoxifen, with 98% vs. 90% being free from local and regional recurrence if they received RT [26]. 

In a subset analysis focusing on inflammatory breast cancer (IBC), older women with more comorbidities were less likely to receive trimodality treatment including RT and had poorer OS, than younger women with fewer comorbidities [30]. The authors suggested that treatment delivery bias may account for younger healthier patients more frequently receiving aggressive, guideline-compliant therapy.

### 4.2. Omission of RT

In some cases, it may be reasonable to omit RT in those with a low chance of recurrence (e.g., ≤2 cm with clear margins, negative axillary lymph nodes, HR^+^), or when risk of toxicity, advancing age and comorbidities outweigh the risk of recurrence [5,10,21,22,23,31]. Older women with a life expectancy <5 years may not derive a survival benefit from RT [12].

An unwarranted fear of toxicity can lead to RT omission even though RT is not considered to be more toxic in older women [10,11,20,21]. RT also may be omitted because of difficulty attending regular clinic visits (e.g., inadequate transportation, accommodation or carer availability), perceived non-compliance with instructions, and limited upper limb mobility (when positioning a patient during RT) [1,3,20]. 

### 4.3. RT and Comorbidities

While there are valid reasons for omitting RT in older women with breast cancer, comorbidities alone should not be the main reason to forgo this therapy. As suggested by several reports in the literature, RT may be considered for most older women when their comorbidities are well managed [12,20,24]. However, in our study, older women with comorbidities were less likely to receive RT than their younger counterparts. 

Older women with breast cancer often have been excluded from clinical trials, resulting in a paucity of evidence about the influence of comorbidities on treatment decisions regarding RT [1,9,10,11,12,20,21,22]. Therapeutic options for older women may not be evidence based, resulting in under treatment with suboptimal outcomes [1,9,11,20,21]. Future studies to assess the effectiveness of RT in patients with comorbidities will benefit by including a representative sample of older women [9,11,12,21]. Analyses of secondary and linked data also can help inform study design [9].

### 4.4. Decision Making Tools

To our knowledge, there is a lack of validated decision-making tools to help clinicians identify which older women will benefit from RT and to assist them in making treatment decisions [22,32]. While some instruments, such as the Comprehensive Geriatric Assessment tool, are useful to measure comorbidities and functional/mental status, they are limited in predicting survival rates in older women with breast cancer [20,21]. An appropriate decision tool should consider the benefits of RT, especially in the context of tumor biology, toxicity, life expectancy, patient preference, quality of life and comorbidities [11,23,33]. Tools that assist older women with breast cancer to make decisions regarding RT also would be valuable.

### 4.5. Strengths and Limitations

Little is known about breast cancer and aging, especially in the context of RT, comorbidities, and biologic subtypes [1,9]. By using NCDB, a large national sample encompassing ~70% of incident cases, we were able to analyse the data by these important characteristics, while controlling for relevant outcome related covariates (e.g., type of therapy, lymph node invasion, margins, race, extent of surgery (BCS/partial mastectomy vs. mastectomy), and tumor size. In comparison, only 25% of new cases are identified through the Surveillance, Epidemiology, and End Results (SEER) program [34]. To our knowledge, this is the first study to simultaneously address trends in RT by age, comorbidity status, and breast cancer biologic subtypes. 

There is no consensus in the literature regarding the age cut-off point for older women, limiting comparison of our results with other studies [35]. Although NCDB is the most comprehensive collection of breast cancer data in the United States, it may underrepresent priority populations such as those lacking comprehensive health insurance [36]. The CCI was reported as three broad categories in NCDB, which may have limited specificity of our results [37]. 

Selection bias may be present as women with more comorbidities may not have progressed to surgery. However, the bias was likely towards the null. Some patients may have refused RT because their tumors were rapidly progressing. While this information was unavailable in NCDB, our analyses were adjusted for factors associated with tumor severity, thus minimizing selection bias. Information on specific systemic therapy also was not available. Again, this limitation likely did not impact results as we were able to stratify analyses by biological subtype, with treatment specificity within these groups. 

Differences in the indication for RT exists by the extent (type) of surgery performed [7,33]. We choose not to limit our study population to quadrantectomy, as RT may be given after mastectomy in the case of 4 or more positive lymph nodes or when the tumor size is >5 cm. Instead, we adjusted for the type of surgery in our multivariable models. 

Cardiovascular comorbidities tend to occur with greater frequencies in older patients and potentially may be exacerbated by RT. However, improvements in RT delivery, which restricts the dose received to the heart, and the long delay between RT and cardiovascular side effects, have minimized this concern [38]. This is supported by our data which did not find important laterality differences between older patients (>65 years) who did and did not receive RT. As observed in the literature, left-sided breast tumors have not been associated with increased mortality risk in patients treated with RT following BCS [38].

Although NCDB is one of the most comprehensive data source for breast cancer in the world, some data fields are unavailable or contain missing values [30]. Consequently, we were unable to assess the impact of specific comorbidities and their severity on treatment decisions regarding RT. The NCDB also did not collect data on disease specific survival. To account for missing data, we use a multistage EM algorithm. Furthermore, variability exists in how data was reported across NCDB sites, limiting the generalizability of our results. Given the large number of comparisons in our study, we cannot rule out that our findings may be attributable to chance. 

## 5. Conclusions

After adjusting for key clinical and demographic factors, we observed that older women with breast cancer and more comorbidities were less likely to receive RT. However, OS was consistently greater for older women who received RT. This was true even in the presence of comorbidities and when the analysis was stratified by biologic subtypes. 

Future studies are needed to better understand the relationship between age, comorbidities and RT treatment decisions and how this impacts on patient outcomes. The personalization of treatment plans regarding RT for older women also is warranted, with emphasis on developing tools to assist clinicians and patients in this process. 

The aetiology of treatment non-compliance and delivery bias are multifactorial in nature [30]. Increasing community, educational, and social efforts aimed at minimizing barriers to RT represents other important steps for future consideration. Additionally, research of older women with breast cancer will benefit from targeted strategies aimed at mitigating the influence of comorbidities in this population [39]. This may include a shorter delivery schedule for RT in the form of an accelerated fractionation plan. 

## Figures and Tables

**Table 1:** Patient characteristics (*n* = 980,381, 2004–2013) ^§^.

Characteristic	Age (Years)			*p* Value ^†^
	<45 *n* (%) Median [IQR]	45–65 *n* (%) Median [IQR]	>65 *n* (%) Median [IQR]	
Overall (*n*)	127,786	517,614	334,981	
**Demographics**				
**Facility type**				<0.0001
Academic/research	52,964 (41)	156,816 (30)	82,258 (25)	
Community	7229 (6)	55,352 (11)	44,994 (13)	
Comprehensive community	59,156 (46)	248,486 (48)	172,210 (51)	
Integrated network	8437 (7)	56,960 (11)	35,519 (11)	
**Great circle distance (miles)**	10 [4]	9 [15]	8 [12]	<0.0001
**Hispanic**	14,507 (11)	37,636 (7)	15,665 (5)	<0.0001
**Insurance**				<0.0001
Medicaid	14,356 (11)	44,324 (9)	6985 (2)	
Medicare	2736 (2)	49,652 (10)	280,882 (84)	
Other government	1624 (1)	6614 (1)	1092 (<1)	
Private	103,805 (81)	403,061 (78)	44,565 (13)	
None	5265 (4)	13,963 (3)	1457 (<1)	
**Income (US $)**				<0.0001
<38,000	17,817 (14)	74,639 (14)	52,688 (16)	
38,000–47,999	25,131 (20)	107,224 (21)	78,190 (23)	
48,000–62,999	34,099 (27)	139,865 (27)	93,572 (28)	
63,000+	50,739 (40)	195,886 (38)	110,531 (33)	
**Race**				<0.0001
Black	18,933 (15)	60,820 (12)	29,177 (9)	
White	99,832 (78)	432,056 (83)	296,153 (88)	
Other	9021 (7)	24,738 (5)	9651 (3)	
**Clinical**				
**Clinical stage (AJCC)**				<0.0001
I	41,433 (32)	224,219 (43)	162,074 (48)	
II	41,488 (32)	130,998 (25)	76,745 (23)	
III	44,865 (35)	162,397 (31)	96,162 (29)	
**Charlson (Deyo) Comorbidity Index**				<0.0001
0	120,259 (94)	455,787 (88)	265,192 (79)	
1	6818 (5)	52,573 (10)	55,730 (17)	
2	709 (1)	9254 (2)	14,059 (4)	
**Differentiation (Grade)**				<0.0001
Well (I)	15,599 (12)	108,904 (21)	85,473 (26)	
Moderately (II)	51,151 (40)	234,551 (45)	164,743 (49)	
Poorly (III)	60,257 (47)	171,836 (33)	83,680 (25)	
Non (IV)	779 (1)	2323 (<1)	1085 (<1)	
**Biologic subtype**				<0.0001
HR^+^/HER_2_^−^	52,857 (41)	251,859 (49)	176,543 (53)	
HR^+^/HER_2_^+^	18,547 (15)	75,850 (15)	47,845 (14)	
HR^−^/HER_2_^+^	24,493 (19)	89,104 (17)	52,700 (16)	
HR^−^/HER_2_^−^	31,889 (25)	100,801 (19)	57,893 (17)	
**Histology (ICD-O-3)**				<0.0001
Infiltrating duct (8500)	104,416 (82)	395,108 (76)	239,639 (72)	
Invasive lobular (8520, 8522)	11,443 (9)	73,191 (14)	54,353 (16)	
Infiltrating duct mixed (8523)	3782 (3)	16,010 (3)	12,236 (4)	
Other	8145 (6)	33,305 (6)	28,753 (8)	
**Lymph node invasion**	37,148 (29)	112,473 (22)	55,017 (16)	<0.0001
**Margins (positive)**	6490 (5)	22,287 (4)	16,612 (5)	<0.0001
**Tumor size (cm)**				<0.0001
≤2	66,117 (52)	326,887 (63)	223,751 (67)	
>2–5	50,259 (39)	159,876 (31)	94,927 (28)	
>5	11,410 (9)	30,851 (6)	16,303 (5)	

^§^ Clinical stage I–III, pathologically confirmed, primary breast cancer. ^†^ Chi-square (categorical) or Kruskal-Wallis H (continuous) test. AJCC: American Joint Committee on Cancer. cm = centimetre. HER = Human epidermal growth factor receptor, HR = Hormone receptor. ICD-O-3: International Classification of Diseases for Oncology, Third Edition. IQR = Interquartile range. NOS: not otherwise specified. US = United States.

**Table 2:** Treatment variables by age (*n* = 980,381, 2004–2013) ^§^.

Treatment	Age (Years)			*p* Value ^†^
	<45 *n* (%) Median [IQR]	45–65 *n* (%) Median [IQR]	>65 *n* (%) Median [IQR]	
**Chemo**	98,703 (77)	292,645 (57)	80,891 (24)	<0.0001
**Endocrine**	78,852 (62)	350,597 (68)	213,620 (64)	<0.0001
**Immuno (only for HER_2_^+^)**	2469 (6)	6839 (4)	2235 (2)	<0.0001
**Neoadjuvant therapy**				
No	120,641 (94)	500,969 (97)	330,098 (99)	<0.0001
Yes	7145 (6)	16,645 (3)	4883 (1)	
Response				
NR	1541 (22)	4146 (25)	1602 (33)	<0.0001
pCR	1412 (20)	2736 (16)	557 (11)	
RD	4192 (59)	9763 (59)	2724 (56)	
**Radiotherapy**				<0.0001
No	53,048 (42)	175,492 (34)	158,469 (47)	
Yes	74,738 (58)	342,122 (66)	176,512 (53)	
Dose (cGy)	5001 [360]	5000 [440]	5000 [540]	<0.0001
4000–5000	37,365 (50)	192,799 (56)	107,659 (61)	<0.0001
>5000–6000	37,373 (50)	149,323 (44)	68,853 (39)	
Lymph nodes treated	25,353 (34)	76,095 (22)	30,014 (17)	<0.0001
**Surgery**				
BCS/partial mastectomy	55,062 (43)	309,460 (60)	213,094 (64)	<0.0001 <0.0001
Mastectomy	72,724 (57)	208,154 (40)	121,887 (36)	
Contralateral	31,007 (43)	57,412 (28)	11,020 (9)	

^§^ Clinical stage I–III, pathologically confirmed, primary breast cancer. ^†^ Chi-square (categorical) or Kruskal-Wallis H (continuous) test. BCS = breast conserving surgery. cGy = centigray NR = No response. HER = Human epidermal growth factor receptor. HR = Hormone receptor. IBC = Inflammatory breast cancer. IQR = Interquartile range. pCR = pathologic Complete response. RD = Residual disease.

**Table 3:** Percentage of patients receiving radiation therapy by age group, comorbidity level and biologic subtype (*n* = 980,381, 2004–2013) ^§^.

Biologic Subtype	Radiation	Charlson/Deyo Comorbidity Index (CCI)															*p* Trend by Age ^†,‡^
		Age <45 Years					Age 45–65 Years					Age >65 Years					
		0 * *n* (%)	I * *n* (%)	II * n (%)	*p* Trend ^†^	aOR ^†,‡^ (95% CI) II vs. 0	0 * *n* (%)	I * *n* (%)	II * *n* (%)	*p* Trend ^†^	aOR ^†,‡^ (95% CI) II vs. 0	0 * *n* (%)	I * *n* (%)	II * *n* (%)	*p* Trend ^†^	aOR ^†,‡^ (95% CI) II vs. 0	
All	N	49,692 (41)	3054 (45)	302 (43)	0.020	1.1 (0.91–1.3)	151,604 (33)	19,849 (38)	4039 (44)	<0.0001	1.3 (1.26–1.41)	120,090 (45)	29,713 (53)	8666 (62)	<0.0001	1.6 (1.5–1.7)	<0.0001
	Y	70,567 (59)	3764 (55)	407 (57)			304,183 (67)	32,724 (62)	5215 (56)			145,102 (55)	26,017 (47)	5393 (38)			
HR^+^ HER_2_^−^	N	19,793 (40)	1327 (44)	140 (42)	0.0029	1.2 (0.92–1.6)	67,842 (31)	9350 (35)	1899 (40)	<0.0001	1.4 (1.2–1.5)	59,307 (43)	15,160 (50)	4498 (59)	<0.0001	1.6 (1.5–1.7)	<0.0001
	Y	29,698 (60)	1705 (56)	194 (58)			152,403 (69)	17,519 (65)	2846 (60)			79,394 (57)	15,041 (50)	3143 (41)			
HR^+^ HER_2_^+^	N	7066 (41)	448 (43)	48 (44)	0.57	1.1 (0.70–1.8)	22,001 (33)	2919 (37)	580 (43)	<0.0001	1.4 (1.2–1.7)	16,677 (44)	4240 (53)	1248 (62)	<0.0001	1.8 (1.6–2.0)	<0.0001
	Y	10,329 (59)	594 (57)	62 (56)			44,690 (67)	4903 (63)	757 (57)			21,131 (56)	3778 (47)	771 (38)			
HR^−^ HER_2_^+^	N	9595 (41)	521 (45)	45 (46)	0.03	1.4 (0.87–2.4)	28,064 (35)	3363 (42)	722 (50)	<0.0001	1.4 (1.2–1.6)	20,581 (49)	4777 (58)	1371 (67)	<0.0001	1.9 (1.5–1.9)	<0.0001
	Y	13,648 (59)	631 (55)	53 (54)			51,622 (65)	4621 (58)	712 (50)			21,861 (52)	3439 (42)	671 (33)			
HR^−^ HER_2_^−^	N	13,238 (44)	758 (48)	69 (41)	0.71	0.80 (0.55–1.2)	33,697 (38)	4217 (43)	838 (48)	0.0011	1.3 (1.1–1.4)	23,525 (51)	5536 (60)	1549 (66)	<0.0001	1.3 (1.2–1.5)	<0.0001
	Y	16,892 (56)	834 (52)	98 (59)			55,468 (62)	5681 (57)	900 (52)			22,716 (49)	3759 (40)	808 (34)			

^§^ Clinical stage I–III, pathologically confirmed, primary breast cancer. * Number corresponds to level of the Charlson (Deyo) Comorbidity Index as coded by NCDB. ^†^ Adjusted for type of therapy (chemo, hormone/endocrine, immune), lymph node invasion, margins, race, extent of surgery (lumpectomy vs. mastectomy), and tumor size. **^‡^** Likelihood ratio trend test. aOR = adjusted odds ratio. HER2 = human epidermal growth factor receptor 2. HR = hormone receptor. N = no. Y = yes. NCDB = National Cancer Database.

**Table 4:** Overall survival and hazard ratios by biologic subtype for women >65 years, (*n* = 334,981, 2004–2013) ^§^.

Biologic Subtype	Radiation	Overall Survival (%)				Hazard Ratio (95% CI)	
		Years				Univariable	Multivariable ^†^
		2	5	8	10		
All	N	94	83	73	67	0.45 (0.441–0.454)	0.53 (0.52–0.54)
	Y	98	91	85	80		
HR^+^ HER_2_^−^	N	95	85	76	71	0.41 (0.40–0.42)	0.49 (0.48–0.51)
	Y	98	93	87	84		
HR^+^ HER_2_^+^	N	94	85	77	74	0.40 (0.39–0.42)	0.47 (0.45–0.49)
	Y	98	93	88	84		
HR^−^ HER_2_^+^	N	93	81	72	67	0.47 (0.46–0.49)	0.54 (0.52–0.56)
	Y	98	91	84	80		
HR^−^ HER_2_^−^	N	92	81	70	63	0.54 (0.52–0.55)	0.62 (0.60–0.64)
	Y	96	89	81	75		

^§^ Clinical stage I-III, pathologically confirmed, primary breast cancer. ^†^ Adjusted for Carlson (Deyo) Comorbidity Index, type of therapy (chemo, hormone/endocrine, immune), lymph node invasion, margins, race, extent of surgery (lumpectomy vs. mastectomy), and tumor size. HER2 = human epidermal growth factor receptor 2. HR = hormone receptor. N = no. Y = yes.
